# Carious Lesion Severity Induces Higher Antioxidant System Activity and Consequently Reduces Oxidative Damage in Children's Saliva

**DOI:** 10.1155/2020/3695683

**Published:** 2020-01-28

**Authors:** Heitor Ceolin Araujo, Ana Cláudia Melo Stevanato Nakamune, Wilson Galhego Garcia, Juliano Pelim Pessan, Cristina Antoniali

**Affiliations:** ^1^Graduate Program in Dental Science, São Paulo State University (UNESP), School of Dentistry, Araçatuba, São Paulo, Brazil; ^2^Department of Pediatric Dentistry and Public Health, São Paulo State University (UNESP), School of Dentistry, Araçatuba, São Paulo, Brazil; ^3^Multicenter Graduate Program in Physiological Sciences, São Paulo State University (UNESP), School of Dentistry, Araçatuba, São Paulo, Brazil; ^4^Department of Basic Sciences, São Paulo State University (UNESP), School of Dentistry, Araçatuba, São Paulo, Brazil

## Abstract

Oxidative stress biomarkers can be found at detectable concentrations in saliva. These salivary biomarkers reflect specific oxidation pathways associated with caries and periodontitis. Our study evaluated the influence of dental caries severity (assessed using the ICCMS™ criteria) on the levels of oxidative stress biomarkers in saliva from children. Unstimulated saliva samples were collected from patients (from one to three years old) in a day care center in Birigui, SP, Brazil, two hours after fasting. Children were divided into four groups (*n* = 30/group), according to caries severity: caries free (group A), early carious lesions (group B), moderate carious lesions (group C), and advanced carious lesions (group D). The following salivary biomarkers were determined: total proteins (TP), measured by the Lowry method; oxidative damage, measured by the TBARS method; total antioxidant capacity (TAC); superoxide dismutase (SOD) enzymatic antioxidant activity; and uric acid (UA) non-enzymatic antioxidant activity. Data were analyzed by ANOVA, followed by the Student-Newman-Keuls test, Pearson and Spearman correlation coefficients, and multivariable linear regression (*p* < 0.05). TP, TAC, SOD enzymatic antioxidant activity, and UA non-enzymatic antioxidant activity increased with caries severity, consequently reducing salivary oxidative damage. It was concluded that higher caries severity increases salivary antioxidant system activity, with consequent reduction in salivary oxidative damage.

## 1. Introduction

Severe Early Childhood Caries (S-ECC) is defined as any sign of dental caries on smooth dental surfaces [1] occurring soon after the first teeth erupt, showing a fast progression when untreated [2]. Early diagnosis of carious lesions leads to less invasive and less traumatic treatments [3] and favors the application of differentiated and more effective treatments in the clinical practice. Based on the well-established and widely used International Caries Detection and Assessment System (ICDAS) [4], the International Classification and Management System for Caries (ICCMS™) enables dentists to integrate and to process information about dental condition, including caries risk, so that they can plan, manage, and review their clinical practice [5].

Saliva, which can be collected in a noninvasive and safe way, has been used as a biological fluid to substitute plasma samples in the diagnosis and prognosis of systemic disease, such as sickle cell anemia in children [6], dementia [7], diabetes [8], chronic kidney disease [9], and oral diseases [10, 11]. In this sense, it is known that saliva of children with S-ECC [12] and saliva of children with caries [13] contain higher protein concentration as compared to saliva of caries-free children. Oxidative stress biomarkers can be found at detectable concentrations in saliva because they are stable in this fluid, so that the levels of these biomarkers in saliva reflect specific oxidation pathways associated with caries and periodontitis (apud [14]).

Physiological metabolism is the main source of free radicals [15], and saliva and its antioxidants are considered regulators of oral cavity redox status under physiological and pathological conditions [16]. Salivary redox status, however, may alter the integrity of oral structures, as normal saliva is exposed to a variety of antioxidants [17, 18] and oxidants which are of extrinsic origin, such as diet and oral bacteria, as well as exposure to ionizing and ultraviolet radiation, smoking, and alcohol [15]. Other sources of reactive oxidative species (ROS) may also include dental materials, restorative treatments [15, 19], and medication use [15].

Studies have reported a positive correlation between salivary protein concentration and salivary total antioxidant capacity (TAC) [12, 20, 21]. Furthermore, saliva of children with S-ECC presents reduced concentration of thiobarbituric acid reactive substances (TBARS) as compared to saliva of caries-free children and of adults (aged 17–50 years) with active carious lesions [12, 22]. Contrarily, superoxide dismutase (SOD) and uric acid (UA) levels are significantly higher in saliva of children with S-ECC as compared to saliva of caries-free children [12], suggesting that reduced oxidative damage in saliva of patients with carious lesions could result from increased enzymatic and nonenzymatic antioxidant system activity. On the other hand, lower glutathione (GSH) concentration in saliva of patients with active carious lesions could indicate a tendency to decreased antioxidant status [23]. These contradictory findings point out that oxidative damage and antioxidant status markers can be modulated by pathogenesis and disease progression.

Considering the complex interplay described above, the aim of the present study was to determine the influence of caries severity (classified according to the ICCMS™ criteria) on the levels of enzymatic and nonenzymatic antioxidants in saliva of children, as well as oxidative damage in saliva of children. We hypothesized that carious lesion severity influences the salivary antioxidant status; that is, in the early stages of carious lesions oxidative damage markers would be increased, but salivary antioxidant systems would be activated in advanced stages in order to reduce oxidative damage.

## 2. Materials and Methods

### 2.1. Patient Selection

The Human Ethics Committee of the School of Dentistry, Araçatuba, São Paulo State University (Universidade Estadual Paulista, UNESP), reviewed and approved the research protocol (CAAE 71063417.4.0000.5420). This research involved children aged between one and three years of age, who were participating in educational and preventive oral health programs at the childcare center Dionisia Miragaia Carmine, in the city of Birigui, São Paulo, Brazil (the city is located in the southeast at latitude 21°17′19^″^ south and longitude 50°20′24^″^ east at an altitude of 450 meters; it has an annual average temperature of 22.1°C and human development index of 0.780).

A meeting was held with the daycare directors and the children's parents or legal guardians prior to the beginning of the study, in which the protocol was explained, and doubts were clarified. Free and informed consent terms were then distributed to all parents/guardians. From the signed informed consent forms returned to the researchers, clinical examinations were performed in 563 children for the determination of the ICCMS™ index, performed by a single calibrated dentist (HCA). Subsequently, 120 children were randomly enrolled in the study (blocking stratification), comprising 30 caries-free children (Group A) and 90 subjects presenting carious lesions at different stages (Groups B, C, and D, 30 subjects/group). Initially, 32 children were selected for the caries-free group (Group A); however, two children were excluded due to noncooperation.

The exclusion criteria were children whose parents/guardians did not provide the signed informed consent form and children presenting carious lesions with more than one ICCMS™ classification. Moreover, any alteration in gingival color and morphology, visible biofilm, previous restorations, and dental eruptions associated with exacerbated inflammatory response observed during the clinical examination were also adopted as exclusion criteria. Besides, systemic diseases, chronic or not, and use of medications were also considered as exclusion criteria.

An experienced pediatric dentist calibrated in the area examined all the children under natural light, with the aid of a WHO probe and mirror; a gauze was used for drying. Children were divided into four groups (*n* = 30/group) according to the ICCMS™ criteria [24]: Group A—caries-free; Group B—initial caries; Group C—moderate caries; and Group D—extensive caries. Sample size was determined on the basis of the study by Silva et al. in 2016 [12], who evaluated oxidative stress markers in saliva samples of caries-free children and in those of children with S-ECC.

### 2.2. Saliva Collection

To minimize the possible influence of recent food/beverage intake, unstimulated saliva was collected between 7:00 a.m. and 8:00 a.m., after fasting for 2 h. Oral hygiene was conducted at home by the parent or guardian, who employed only water and toothbrush without fluoride products. In cases of adverse event, in which saliva collection was difficult (intense crying, lack of cooperation), the procedure was interrupted, and it was not attempted again. Saliva samples were collected with the Salivette® cotton swab, which was positioned and maintained in the sublingual space for 5 min. The collected samples were kept on ice. Then, the samples were centrifuged at 5500 × *g* for 10 min, and the supernatants were divided into four aliquots (100–250 *μ*L) and stored at -80°C until analysis [25].

All biochemical analyses were performed by colorimetric reactions. Samples were placed in 96-well plates, except in the case of SOD analysis (using only the first well of a 24-well plate, due to its kinetic reaction) and read in a PowerWave 340 BioTek reader.

### 2.3. Salivary Flow

Salivary flow was determined by the total value of each sample collected (mL) during 5 minutes, and the results were expressed in mL/min.

### 2.4. Total Protein Concentration Determination

Protein was quantified in 20 *μ*L saliva aliquots using the method by Lowry et al. in 1951 [26]. A standard curve for different bovine serum albumin concentrations, ranging from 0.02 to 0.08 mg/mL, was employed. When the concentration in the sample was above the upper point of the standard curve, the sample was diluted and a new determination was made. Absorbance values were determined at 660 nm. Results are expressed as mg/dL.

### 2.5. Substances Reactive to Thiobarbituric Acid (TBARS)

The oxidative damage was evaluated by the TBARS method, which determines the substances reactive to thiobarbituric acid. TBARS was determined as described by Buege and Aust in 1978 [27]. Trichloroacetic acid (10% *w*/*v*) was added to 125 *μ*L of saliva, which acidified the solution and caused the proteins to precipitate. The mixture was centrifuged (1000 × *g*, 3 min), thiobarbituric acid (TBARS, 0.67% *w*/*v*) was added to the supernatant, and the solution was kept in a water bath at 100°C for 15 min. After reaching room temperature, the absorbance was read at 535 nm; a molar absorption coefficient of 1.56 × 10^5^ M^−1^ cm^−1^ was employed. Results are expressed in nmol/L and in nmol/L/mg of protein.

### 2.6. Salivary Total Antioxidant Capacity (TAC)

Salivary TAC was evaluated by the antioxidant power of iron reduction (FRAP assay), as previously standardized by Benzie and Strain in 1996 [28]. This method is based on the reduction of the ferric complex tripyridyl triazine (Fe^3+^ TPTZ), which forms Fe^2+^ in acidic medium. A 15 *μ*L saliva aliquot was used. Absorbance was determined at 595 nm; a standard ferrous sulfate curve with values ranging from 20 to 260 *μ*mol/L FeSO_4_ was employed. Results are expressed in *μ*mol/L FeSO_4_ and in *μ*mol/L FeSO_4_/mg of protein.

### 2.7. Uric Acid (UA)

A commercial kit (Labtest Diagnóstica SA, MG, Brazil) was used to determine UA in 20 *μ*L of saliva by means of the enzymatic method of Trinder. The manufacturer's instructions were followed. Results are expressed as mg/mL and as mg/mL/mg of protein.

### 2.8. Superoxide Dismutase (SOD) Activity

On the basis of pyrogallol autoxidation inhibition, SOD activity was determined in saliva by the method of Maklund in 1985 [29]. A 20 *μ*L aliquot of saliva was previously diluted (1 : 10, *v*/*v*). Absorbance was read at 420 nm. Five readings were taken every 1 min. The amount of enzyme that was necessary to reduce pyrogallol autoxidation by 50% was considered as a unit of enzymatic activity. Results are expressed in UE/mL and in UE/mL/mg of protein.

### 2.9. Statistical Analysis

Data are expressed as mean ± SD (standard deviation). Data showed normal distribution (Shapiro-Wilk's test) and was submitted to ANOVA and Student-Newman-Keuls' posttest. The relationship between the dependent variables (TBARS, TAC, SOD, and UA) was examined by the Pearson correlation coefficient, and the nonparametric Spearman correlation test was performed for correlations between groups (ICCMS™) and dependent variables (GraphPad, version 5.0, GraphPad Software Corporation, La Jolla, CA, USA). Values of *r* between 0.50 and 0.69, 0.70 to 0.89, and >0.90 were considered as moderate, strong, and very strong correlations, respectively [30]. The multivariable linear regression test was applied to verify the influence of groups (ICCMS™), age, and number of carious lesions on oxidative damage (Statistica 13.0 software). Differences were considered significant when *p* < 0.05.

## 3. Results

Gender was fairly distributed within each group, with boys corresponding to 46.6% in Groups A and C and 50% in Groups B and D. The age of the children progressively increased from Group A to Group D. The number of erupted teeth also increased progressively from Groups A to C, but there was no difference between Groups C and D. The number of carious lesions was not significant among the Groups B, C, and D. Salivary flow rate was not significantly different among the groups (Table 1).

**Figure 1:**
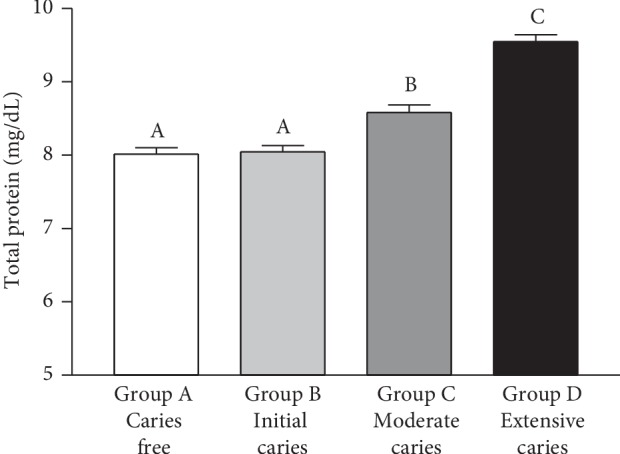
Total protein (mg/dL) in saliva samples of caries-free children (Group A) and in those with caries at different stages (Groups B-D). Bars represent the mean ± SD. Equal letters represent statistical similarity (*p* > 0.05) and different letters mean statistical differences (*p* < 0.001).

Total salivary protein concentration was higher (*p* < 0.001) in Group D as compared to the other groups. Higher concentrations were observed in Group C than in Groups A and B (*p* < 0.001), with no significant differences between Groups A and B (*p* > 0.05) (Figure 1). There was a positive but moderate correlation between the amount of salivary proteins and caries severity (Spearman's *r* = 0.7084, *p* < 0.0001).

**Figure 2:**
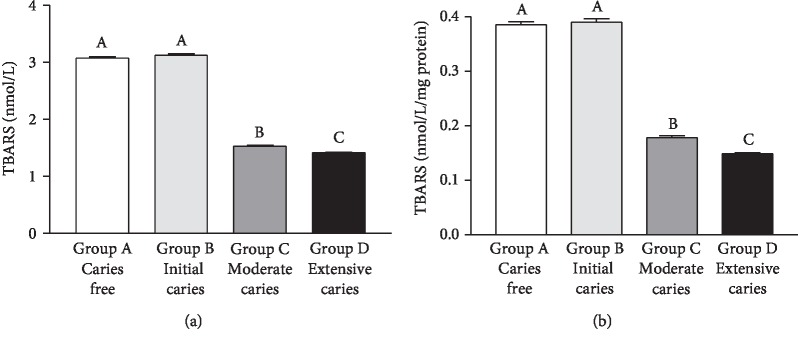
Concentration of 2-thiobarbituric acid-reactive substances (TBARS) (in nmol/L for the graph in (a) and in nmol/L/mg protein for the graph in (b)) in the saliva samples of caries-free children (Group A) and in those with caries at different stages (Groups B-D). Bars represent the mean ± SD. Equal letters represent statistical similarity (*p* > 0.05) and different letters mean statistical differences (*p* < 0.001).

Salivary MDA concentration (Figure 2(a)) or salivary MDA concentration standardized by mg of total protein (Figure 2(b)) was lower (*p* < 0.0001) in Group D than in the other groups. Such levels were lower in Group C than in Groups A and B, with no significant differences between Groups A and B (*p* > 0.05). There was a strong negative correlation between caries severity and salivary MDA values (Spearman's *r* = −0.8570, *p* < 0.0001). MDA values and total salivary proteins correlated negatively, but moderately (Table 2).  Table 3 presents the multivariate linear regression analysis results for oxidative damage (MDA concentration, dependent variable) and caries severity (ICCMS™), number of lesions, and age (independent variables). Only caries severity and the number of lesions were shown to influence MDA concentrations.

**Figure 3:**
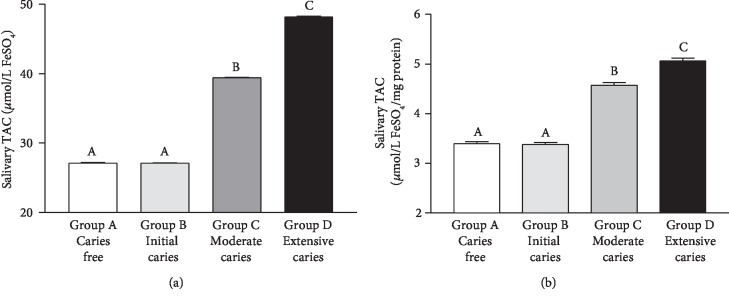
Total Antioxidant Capacity (TAC) (in *μ*mol/L FeSO_4_ for the graph in (a) and in *μ*mol/L FeSO_4_/mg protein for the graph in (b)) in saliva samples of caries-free children (Group A) and in those with caries at different stages (Groups B-D). Bars represent the mean ± SD. Equal letters represent statistical similarity (*p* > 0.05) and different letters mean statistical differences (*p* < 0.001).

Salivary TAC (Figure 3(a)) or salivary TAC normalized by mg protein concentration (Figure 3(b)) was higher (*p* < 0.001) in Group D than in the other groups. Higher levels were observed in Group C than in Groups A and B (*p* < 0.001), without significant differences between Groups A and B (*p* > 0.05). A positive and strong correlation was observed between caries severity (ICCMS™) and salivary TAC (Spearman's *r* = 0.8425, *p* < 0.0001). In addition, there was a very strong negative correlation between TAC and salivary MDA (Table 2).

**Figure 4:**
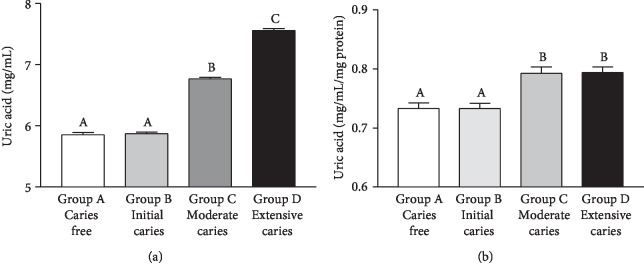
Uric acid (UA) concentration (in mg/mL for the graph in (a) and in mg/mL/mg protein for the graph in (b)) in saliva samples of caries-free children (Group A) and in those with caries at different stages (Groups B-D). Bars represent the mean ± SD. Equal letters represent statistical similarity (*p* > 0.05) and different letters mean statistical differences (*p* < 0.001).

Salivary UA concentration was increased (*p* < 0.0001) in Group D as compared to the other groups. In Group C, UA concentration was higher than in Groups A and B (*p* < 0.001), without significant difference between Groups A and B (*p* > 0.05) (Figure 4(a)). When salivary UA were normalized by mg protein concentration, values were increased (*p* < 0.0001) in Groups C and D as compared to Groups A and B; however, there were no significant differences between Groups C and D (*p* > 0.05) (Figure 4(b)). There was a positive, weak correlation between UA/mg protein values and groups (Spearman's *r* = 0.4659, *p* < 0.0001). Moreover, UA/mg of protein values and salivary TAC correlated strongly and positively, whilst UA and salivary MDA correlated weakly and negatively (Table 2).

**Figure 5:**
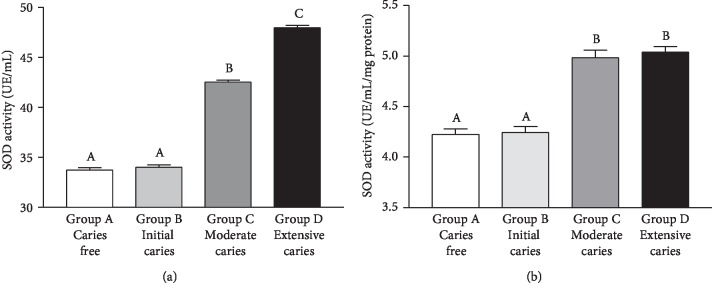
Superoxide dismutase (SOD) activity (in UE/mL for the graph in (a) and in UE/mL/mg protein for the graph in (b)) in saliva samples of caries-free children (Group A) and in those of with caries at different stages (Groups B-D). Bars represent the mean ± SD. Equal letters represent statistical similarity (*p* > 0.05) and different letters mean statistical differences (*p* < 0.001).

SOD activity was significantly higher (*p* < 0.001) in Group D than in the other groups. In Group C, SOD activity was greater than in Groups A and B (*p* < 0.001), without significant differences between Groups A and B (*p* > 0.05) (Figure 5(a)). When normalized, SOD activity/mg protein was higher (*p* < 0.001) in Groups C and D than in the Groups A and B, but there were no differences between Groups A and B (*p* > 0.05) and C and D (*p* > 0.05) (Figure 5(b)). There was a positive and strong correlation between caries severity and salivary SOD activity (Spearman's *r* = 0.7320, *p* < 0.0001). SOD activity correlated moderately negatively with salivary oxidative damage (Table 2).

## 4. Discussion

The development of clinical diagnostic criteria that consider carious lesions at different stages of tissue compromising has allowed a better comprehension of dental caries as a progressive disease, in addition to the establishment of preventive and therapeutic measures depending on the degree of tissue damage. Considering the lack of studies assessing the relationship between caries severity and salivary oxidative stress in children, the present study assessed the oxidative stress biomarkers in saliva of children presenting carious lesions at different stages, according to the ICCMS™ criteria. The present results showed that the concentrations of total protein and oxidative stress biomarkers in saliva of children with initial carious lesions (*i.e.*, without cavitation) were not significantly different from caries-free children. On the other hand, the higher the lesion severity, the higher the concentrations of salivary proteins and oxidative stress biomarkers. Together, these findings may contribute to future clinical and biochemical studies in the area of cariology.

Salivary protein concentrations in children with cavitated carious lesions in enamel and dentin (Groups C and D, respectively) resemble the protein concentration previously quantified in saliva of children with S-ECC (grouped without taking caries severity into account) [12], but are higher than the protein concentration detected in caries-free children. Jurczak et al. in 2017 [31] obtained higher values in saliva of children with cavitated lesions (ICDAS = 5‐6, protein concentration = 1.86 mg/mL) than in saliva of children without cavitated carious lesions (ICDAS = 1‐2, protein concentration = 1.79 mg/mL). Nonetheless, both values were higher than the protein concentration measured in caries-free children (1.65 mg/mL). It must be pointed out that salivary protein concentrations quantified in the different groups included in the study by Jurczak et al. in 2017 [31] were almost two-fold higher than in the present study. This is probably because Jurczak et al. in 2017 used chew-stimulated saliva samples, which are associated with increased protein values as compared to nonstimulated saliva [31]. In contrast, Tulunoglu et al. in 2006 [13] reported that salivary protein values in children aged 7–10 years old (6.5 mg/mL) did not significantly differ from salivary protein values in caries-free children (3.5 mg/mL). Furthermore, higher protein concentrations were detected in saliva of 7-10 year-old children than in 1-3 year-old ones, which is in line with previous observations that salivary protein concentration increases with children's age [13].

Caries severity was shown to increase the amount of salivary proteins, as these variables were positively correlated. Carious lesions in dentin are associated with mineral and organic matrix degradation by the activity of collagenolytic enzymes, mainly matrix metalloproteinases (MMPs), and high MMP-8 concentrations have been found in saliva of individuals with carious lesions in dentin [32]. In addition, proteomic analysis of saliva of individuals with caries lesions revealed greater expression of antimicrobial proteins and degradation-resistant protein complexes such as mucin 7, mucin 5B, cystatin S, cystatin SN, rich protein, proline base saline, and histatin 11 [33].

TBARS assay was used as a rapid screening procedure [34], since it is one of the most classical methods for determining oxidative damage in tissues and homogenates [35, 36], plasma [37], salivary glands [38], and also in saliva [12, 25]. This study demonstrated that salivary TBARS concentrations gradually decreased in children with cavitated lesions (enamel and dentin) as compared to caries-free children or those with initial lesions. Our results corroborate previously obtained data showing that the TBARS concentration in saliva of children with E-SCC (0.0019 *μ*mol/L/mg of protein) is lower than in saliva of caries-free children (0.0039 *μ*mol/L/mg of protein). However, recent studies [39, 40] have reported controversial results: salivary TBARS concentrations in children aged six years and in patients aged 15–19 years with caries lesions (0.26 *μ*mol/L and 0.71 nmol/mL, respectively) are increased as compared to caries-free patients. Methodological differences may account for the discrepancies between our results and literature data. It is noteworthy that TBARS values were not normalized by the salivary protein concentration in neither of the aforementioned literature studies. Because the presence and progression of dental caries raise the salivary protein concentration, the TBARS concentration values must be normalized by the amount of salivary protein found in each group. Caries severity and salivary TBARS values are strongly and negatively correlated, which reinforces our results. In addition, multivariate linear regression shows that caries severity is the variable associated with salivary oxidative damage. Taken together, our data demonstrate that carious lesion progression reduces salivary oxidative damage [39, 40].

The mechanisms that reduce salivary oxidative damage have been recently evaluated, and TAC assessment provides information on the balance between oxidant and antioxidant systems, *i.e.*, a high amount of antioxidant contributes to reduce oxidative damage [41]. Salivary TAC was shown to increase with carious lesion severity (dentin or enamel) in the present study. TAC in saliva of S-ECC children [12] and in children (mean age 2.74 years) with cavitated carious lesions [31] have been reported to be higher than in caries-free children or in those with noncavitated lesions. However, in patients with carious lesions, salivary TAC tends to decrease as compared to caries-free patients [42]. Surprisingly, individuals aged 15–19 years old with active caries lesion cavities present similar salivary TAC (0.17 mol/mL) compared to those without carious lesions (0.17 mol/mL) [31], suggesting that age may be an important factor in salivary TAC quantification. It was noteworthy that increased salivary TAC of children with cavitated lesions (enamel and dentine) was negatively correlated with oxidative damage rates, which reinforces the suggestion that salivary TAC is a marker for dental caries activity in children, as previously proposed by Krumar et al. in 2015 [43].

TAC reflects the combined effects of antioxidants (mainly nonenzymatic) present in plasma and body fluids [44]. Given that TAC is related to nonenzymatic antioxidant system activity, the UA antioxidant activity was also evaluated in the present study, as UA is an efficient antioxidant that eliminates reactive species from plasma [45]. As demonstrated by a positive correlation between the UA in plasma and saliva [46–48], saliva seems to be a good substitute of blood for the evaluation of this biomarker. The only study that quantified this antioxidant in saliva of children with caries observed higher salivary UA values (7.05 mg/mL) as compared to caries-free children (5.02 mg/mL) [12]. Here, saliva of children with carious lesions in dentin presented the highest UA value, followed by saliva of children with carious lesions in enamel. UA values normalized by mg total protein (Figure 3(b)) showed an increase in nonenzymatic antioxidant activity at the most severe caries stages, despite that no significant differences were observed between Group C and Group D. Moreover, a positive but weak correlation was observed between salivary UA/mg protein values and caries severity. Together, these results suggest that other nonenzymatic antioxidants besides UA might have contributed to the progressive increase in TAC and to the progressive reduction in MDA in saliva of children in Groups C and D. Overall, our results indicate that salivary oxidative damage is lower in children with carious lesions due to the higher salivary UA antioxidant effect, corroborating the suggestion that increased UA would be an adaptive protection response against excess reactive species [45].

The contribution of enzymatic antioxidant activity to oxidative damage in saliva of children with caries lesions has also been evaluated. To this end, the activity of SOD, which is the enzymatic antioxidant system that is considered as the body's first line of defense against reactive species, has been investigated [49]. SOD catalyzes the superoxide anion dismutation into oxygen and hydrogen peroxide [50], so that the superoxide anion decreases the nitric oxide (NO) bioavailability [51, 52]. NO is one of the defense mechanisms of the oral cavity against bacterial multiplication (apud [53]). In patients with a mean age of 21 years, there is an increase in salivary NO in individuals with dental caries and poor hygiene [53]. Therefore, NO production may be a mechanism of host defense when dental caries increases or oral hygiene is impaired. Salivary SOD activity in children with S-ECC aged 0–3 years is higher than in caries-free children [12]. Increased salivary SOD activity has also been observed in patients aged 25–50 years with active caries as compared to caries-free patients [54]. In the present study, caries severity was shown to increase salivary SOD activity, which in turn contributes to gradual reduction in salivary oxidative damage in children with carious lesions in enamel and dentin. The normalized SOD activity by mg total protein showed an increase in enzymatic antioxidant activity in the most severe caries stages, but without significant differences between Group C and Group D. However, a positive and strong correlation between caries severity and salivary SOD activity was observed in our study. Moreover, SOD activity correlated moderately negatively with salivary oxidative damage. The progressive increase in salivary proteins in Groups C and D indicate that proteins other than SOD may contribute to the progressive reduction of MDA. Our study suggests that increased SOD activity in saliva of children with active carious lesions could be a host defense mechanism against dental caries, *i.e.*, higher SOD activity in saliva increases NO bioavailability.

On the basis of the results presented herein, we can conclude that caries severity has a direct effect on the activity of antioxidant systems and salivary TAC, which gradually reduces salivary oxidative damage.

## Figures and Tables

**Table 1:** General characteristics of children by group.

	Group A		Group B		Group C		Group D	
Age (months)	17.8 ± 4.5^a^		23.6 ± 5.6^b^		32.6 ± 4.5^c^		35.0 ± 2.8^d^	
Gender	Boys	Girls	Boys	Girls	Boys	Girls	Boys	Girls
	46.6%^a^	53.4%^a^	50%^a^	50%^a^	46.6%^a^	53.4%^a^	50%^a^	50%^a^
Number of erupted teeth	12.7 ± 3.8^a^		17.4 ± 40^b^		19.8 ± 0.5^c^		19.9 ± 0.3^c^	
Number of lesions ± SD	0		1.93 ± 0.94^a^		1.83 ± 0.87^a^		2.57 ± 1.10^a^	
Salivary flow (mL/min)	0.187 ± 0.017^a^		0.180 ± 0.013^a^		0.185 ± 0.013^a^		0.181 ± 0.015^a^	

Similar superscript letters on the same line mean statistical similarity (*p* > 0.05). Different superscript letters on the same line mean statistical differences (*p* < 0.05).

**Table 2:** Pearson's correlation coefficients (*r* values) among the dependent variables: TBARS (substances reactive to thiobarbituric acid), TAC (total antioxidant capacity), UA (uric acid), and SOD (superoxide dismutase).

	TAC (mmol/L FeSO_4_/mg protein)	UA (mg/mL/mg protein)	SOD (UE/mL/mg protein)	
TBARS (mg/mL/mg protein)	-0.85	-0.35	-0.65	*p* < 0.0001
TAC		0.72		

**Table 3:** Multivariate linear regression analysis of factors associated with oxidative damage.

	Coefficient	Standard deviation	*t*	*p*	VIF	*R* ^2^ adjusted
Groups	-0.000986	0.0000737	-13.39	<0.001	3.808	0.832
No. of lesions	0.000219	0.000045	4.872	<0.001	1.81	
Age (in months)	-0.0000132	0.00000887	-1.486	0.14	2.949	

VIF=collinearity; *t* = force of the equation.

## Data Availability

The individual data obtained from each patient were not made available by the authors.
